# Aligned Hollow Silicon Nanorods Containing Ionic Liquid Enhanced Solid Polymer Electrolytes with Superior Cycling and Rate Performance

**DOI:** 10.1002/advs.202411437

**Published:** 2024-11-21

**Authors:** Xinglong Gao, Zhong Zheng, Yifan Pan, Shuyi Song, Zhen Xu

**Affiliations:** ^1^ Hubei Key Laboratory of Modern Manufacturing Quantity Engineering School of Mechanical Engineering Hubei University of Technology Wuhan Hubei 430068 China; ^2^ Xinjiang Key Laboratory of High Value Green Utilization of Low‐Rank Coal School of Physics and Materials Science Changji University Changji Xinjiang 831100 China; ^3^ School of Energy and Power Engineering Shandong University Qingdao Shandong 266100 China

**Keywords:** hollow silicon nanorods, ionic liquid, lithium‐ion conductivity, orientation alignment, solid‐state lithium battery

## Abstract

The low lithium‐ion conductivity of polyethylene oxide (PEO)‐based polymer electrolytes limits their application in solid‐state lithium batteries and related fields. Here, ionic liquids (ILs) are injected into hollow silicon nanorods (HSNRs) to prepare a composite solid polymer electrolyte (CSPE) with aligned HSNRs containing ILs (F‐ILs@HSNRs). Applying a magnetic field promoted uniform dispersion and orientation of F‐ILs@HSNRs in CSPE. The addition of F‐ILs@HSNRs reduced PEO crystallinity and formed Li^+^ transport pathways at the F‐ILs@HSNRs/PEO interface. Calculations and multi‐physics simulations reveal that ILs within F‐ILs@HSNRs contribute most to lithium‐ion conduction, followed by the F‐ILs@HSNRs/PEO interface. When F‐ILs@HSNRs are arranged perpendicular to the electrodes, the CSPE exhibits the shortest Li^+^ migration pathways, resulting in stable and efficient lithium‐ion conduction. The conductivity (2.14 × 10^−4^ S cm^−1^) and lithium‐ion migration number t_Li+_ (0.307) are the highest, being 125 times and 184% higher, respectively, than those of PEO‐LiTFSI, when compared to CSPEs with randomly arranged or parallel‐aligned F‐ILs@HSNRs. Furthermore, Li|CSPE|Li batteries and LiFePO_4_|CSPE|Li batteries display stable cycling for over 2000 h, with coulombic efficiency approaching 100%. Excellent electrochemical reversibility is also confirmed in the rate performance test.

## Graphical abstract

Ionic liquid (IL) is injected into hollow silicon nanorods (HSNRs) to prepare composite solid polymer electrolyte (CSPE) with aligned HSNRs containing ILs (F‐ILs@HSNRs). CSPE with F‐ILs@HSNRs perpendicular to electrodes has the shortest Li^+^ fast migration pathways, with 125 times and 184% improvements in conductivity and lithium‐ion migration number. Its batteries exhibit excellent electrochemical reversibility, with coulomb efficiency approaching 100%.

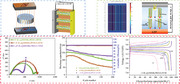

## 1 Introduction

Solid‐state lithium batteries based on solid electrolytes have garnered significant attention for their potential to address battery safety concerns. With ongoing advancements in the energy industry and increasing safety requirements in practical applications, this area of research has seen growing interest. Inorganic solid electrolytes currently offer high lithium‐ion conductivity (10^−3^–10^−4^ S cm^−1^); however, they are often incompatible with electrode interfaces.^[^ ^1^, ^2^, ^3^ ^]^ Conversely, polymer electrolytes, particularly polyethylene oxide (PEO)‐based electrolytes, are lightweight, flexible, and exhibit good compatibility between the electrolyte and electrodes, while providing high operational safety. However, their relatively low lithium‐ion conductivity at 25 °C (10^−6^–10^−7^ S cm^−1^) and high manufacturing costs limit their widespread application.^[^ ^4^, ^5^, ^6^ ^]^ Organic‐inorganic composite solid electrolytes, which integrate the high conductivity of inorganic electrolytes with the flexibility of organic electrolytes, have emerged as a key research focus.^[^ ^7^, ^8^, ^9^ ^]^ However, the lithium‐ion conductivity of these composite electrolytes has not reached the levels required for practical use.

The inorganic phases in composite solid polymer electrolytes (CSPEs) are generally dispersed in the polymer matrix in the form of micro‐ and nano‐scale particles. These inorganic fillers can be divided into inert and active fillers according to whether they can conduct lithium‐ion electrons.^[^ ^10^ ^]^ Typical inert fillers include SiO_2_, Al_2_O_3_, and TiO_2_,^[^ ^11^ ^]^ which mainly improve the mobility of polymer chains by hindering their rearrangement and crystallization through the anchoring effect. Inert fillers have limited ability to enhance lithium‐ion transport performance (typically up to 10^−5^ S cm^−1^). The active fillers are mainly various inorganic solid electrolyte materials such as oxide solid electrolyte garnet‐type Li_7_La_3_Zr_2_O_12_ (LLZO) (with a conductivity of 10^−4^ ‐ 10^−3^ S cm^−1^), and sulfide solid electrolytes such as Li_10_GeP_2_S_12_ (with a conductivity of 10^−2^ S cm^−1^).^[^ ^12^, ^13^ ^]^ In addition to enhancing ion conductivity by reducing the crystallinity of the polymer phase, active fillers can also provide ion transport pathways with high conductivity. These active particles can improve the lithium‐ion conductivity of CSPE (up to 10^−4^ S cm^−1^).

LLZO solid electrolyte nanowires, a notable example of oxide solid electrolytes, have attracted considerable attention owing to their ability to form a 3D continuous ion‐conducting network within polymers.^[^ ^14^ ^]^ For example, Zhang et al. utilized a cellulose template method to construct a 3D LLZO network, achieving a room temperature lithium‐ion conductivity of 1.37 × 10^−4^ S cm^−1^ in composite solid polymer electrolytes.^[^ ^15^, ^16^ ^]^ However, 1D inorganic solid electrolyte fabrication methods are often expensive and inefficient.^[^ ^17^, ^18^ ^]^ To address this, Cheng et al. used high‐conductivity LLZO nanowires combined with PEO polymer electrolytes to create a 3D LLZO network, raising the electrolyte conductivity to 2.37 × 10^−4^ S cm^−1^ at room temperature.^[^ ^19^, ^20^ ^]^ Despite these improvements, the complex and expensive production process, along with low yields, continues to hinder the industrial viability of LLZO.^[18,^ ^21^, ^22^ ^]^

Natural clay minerals hold considerable promise for use in organic–inorganic composite solid electrolytes due to their abundance of pores and channels, high specific surface area, porous structure, excellent adsorption properties, and low cost. Sepiolite ((Si_12_) (Mg_8_) O_30_ (OH)_4_ (OH_2_)_4_·8H_2_O), a magnesium (Mg) silicate clay mineral, is a naturally abundant fibrous crystal with unique channels that permeate its structure and a large surface area. Sepiolite hollow silicon nanorods (HSNRs) have a channel cross‐sectional area of 0.36 × 1.06 nm and a theoretical surface area of up to 900 m^2^·g^−1^.^[^ ^23^, ^24^ ^]^ Han et al. employed sepiolite nanorods to create PEO‐based organic–inorganic composite solid electrolytes, showing that the introduction of these inorganic nanofillers effectively enhances the mechanical properties of the electrolyte.^[^ ^25^ ^]^ However, the weak conductivity of sepiolite nanorods limits lithium‐ion migration, leading to low energy density and significantly restricting their application.

Ionic liquids (ILs) are green solvents with low vapor pressure and wide electrochemical windows. They are non‐volatile, non‐flammable, and exhibit high compatibility with lithium salts and various organic solvents. ILs exhibit high conductivity, generally ranging from 10^−2^ to 10^−3^ S cm^−1^,^[^ ^26^, ^27^ ^]^ and are widely used in electrochemistry, adsorption separation, and catalysis.

To fully leverage the excellent conductivity of ILs, improve the conductivity of sepiolite nanorods, and shorten lithium‐ion migration pathways, this study introduces ILs into HSNRs and strategically aligns them perpendicular to the electrodes. HSNRs with confined ILs significantly reduce the consumption of high‐cost ILs compared to their free dispersion in a PEO polymer matrix.^[^ ^28^, ^29^ ^]^ Additionally, orienting HSNRs containing ILs perpendicular to the electrodes shortens lithium‐ion migration pathways, enabling stable and efficient ion transport.

Herein, to enhance the electrochemical performance of solid‐state lithium batteries at a low cost, a highly conductive (10^−2^ S cm^−1^) IL, and an inexpensive 1D hollow material, natural sepiolite, were selected.^[^ ^30^ ^]^ HSNRs were prepared from natural sepiolite via a microwave‐assisted acid activation method and magnetically modified through chemical precipitation. The IL was injected into and confined within the hollow HSNR channels using a supercritical fluid process. With magnetic field alignment, a CSPE with aligned HSNRs containing ILs was successfully constructed and used to assemble solid‐state lithium batteries. This study investigates the effects of CSPE composition and structure on the conductivity, electrochemical performance, cycling stability, and rate performance of CSPE‐based lithium batteries. The mechanisms governing the behavior of HSNRs and CSPEs were explored through calculations and multi‐physics field simulation analysis.

## 2 Experimental Section

### 2.1 Preparation of CSPE with Aligned HSNRs Containing ILs

#### 2.1.1 Materials

Original sepiolite ore was sourced from Hunan Xiangtan Yuanyuan Sepiolite New Material Co., Ltd. Lithium bis (trifluoromethanesulfonyl) imide (LiTFSI, 99.0%) and polyethylene oxide (PEO, 99.0%) were obtained from Aladdin Industrial Corporation. Ionic liquid 1‐ethyl‐3‐methylimidazolium bis (trifluoromethanesulfonyl) imide (EMI TFSI) was procured from Moni Chemical Technology (Shanghai) Co., Ltd. Ferrous sulfate (FeSO_4_·7H_2_O, 99.0%), ferric chloride (FeCl_3_·6H_2_O, 99.0%), hydrochloric acid (HCl, 36.0–38.0%), and anhydrous ethanol (CH_3_CH_2_OH, 99.7%) were supplied by National Pharmaceutical Reagent Co., Ltd.

#### 2.1.2 Microwave‐Assisted Acid Activation of Sepiolite

Pristine sepiolite ore (10.0 g) was placed in a three‐necked flask, and hydrochloric acid solution (2 mol L^−1^) was added at a liquid‐to‐solid ratio of 10:1 (mL g^−1^). The mixture was processed in a microwave workstation at a power of 1000 W, stirring speed of 500 R/min, and temperature of 80 °C for 2 h. The treated sepiolite was then washed with anhydrous ethanol and deionized water, dried at 105 °C, and ground to produce acid‐activated sepiolite, herein referred to as HSNRs.

#### 2.1.3 Preparation of Magnetized HSNRs

FeSO_4_·7H_2_O (1 g) and FeCl_3_·6H_2_O (1.5 g) were mixed, followed by the addition of 150 mL of deionized water and 6 g of HSNRs. The mixture was heated to 40 °C and maintained at this temperature in a water bath with agitation for 20 min. NH_3_·H_2_O was then added to the flask until the pH reached ≥ 10. The solution was stirred for an additional 12 h and cooled to 25 °C. The resulting powder was washed with anhydrous ethanol and deionized water, vacuum dried at 105 °C for 12 h, and ground to obtain Fe_3_O_4_‐attached HSNRs; herein referred to as F‐HSNRs.

#### 2.1.4 Injection of ILs into F‐HSNRs

Magnetized HSNRs (1 g), ILs (1 g), and anhydrous ethanol (6 g) were added into a reactor. Supercritical CO_2_ fluid was slowly injected into the sealed reactor, and the reaction was conducted at 12 MPa and 60 °C for 6 h.^[^ ^31^, ^32^, ^33^, ^34^ ^]^ The resulting F‐HSNRs containing confined ILs were herein referred to as F‐ILs@HSNRs.

#### 2.1.5 Preparation of CSPE

**Figure 1:**
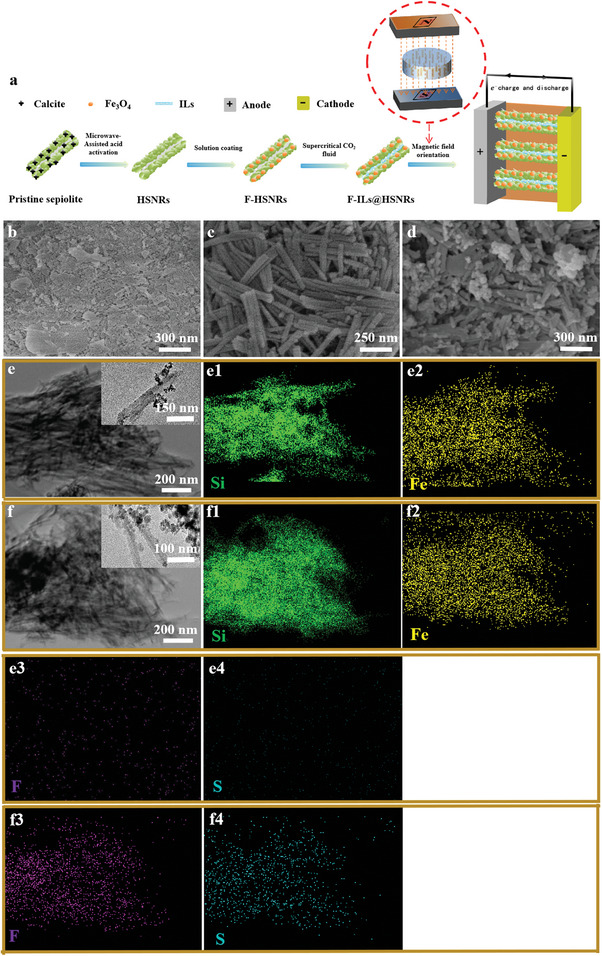
a) Schematic illustration of the preparation of a CSPE battery with aligned HSNRs containing ILs. SEM images of b) pristine sepiolite ore, c) acid activated, and d) magnetized samples. TEM images and elemental mapping of sepiolite after e–e4) acid activation and magnetization, and f–f4) IL injection.

PEO (M_w_ = 3 × 10^6^) and LiTFSI were dissolved in acetonitrile, and F‐ILs@HSNRs were added at a concentration of 15 wt%, with an EO/Li molar ratio of 16:1.^[^ ^35^ ^]^ This ratio was determined based on relevant experimental data (Supporting Information, see discussion for Figure S1, Supporting Information). The mixed solution was cast into a cylindrical cavity of a polytetrafluoroethylene (PTFE) mold with an inner diameter of 16 mm. A strong magnetic field was applied to the PTFE mold filled with the mixed solution at room temperature for 24 h using a neodymium magnet, and the direction of the magnetic field N‐S was controlled to be perpendicular to or parallel to the liquid surface of the solution, as illustrated in **Figure** **1**a. The mixed solution was then dried at 50 °C under vacuum for 24 h to obtain a cylindrical CSPE with magnetically aligned F‐ILs@HSNRs.

### 2.2 Assembly of Solid‐State Lithium Batteries

The active material (LiFePO_4_), conductive carbon black (Super P), PEO, and LiTFSI salt were uniformly mixed at a mass ratio of 60:12:20:8 at 600 rad min^−1^.^[^ ^36^ ^]^ The slurry was evenly coated on aluminum foil using a blade and dried at 60 °C in a vacuum oven. Subsequently, a cathode sheet with a diameter of 10 mm was prepared by punching. The active material loading in the cathode was ≈1.3 mg cm^−2^. LiFePO_4_|CSPE|Li batteries were assembled using LiFePO_4_ as the cathode, metallic lithium as the anode, and CSPE as the solid‐state electrolyte. When assembling the batteries, the electrode sheets were perpendicular to the circular upper surface of the cylindrical CSPE. Lithium symmetrical batteries were assembled in the same way. CSPEs with ILs@F‐HSNRs arranged randomly, parallel, and perpendicular to the electrodes and labeled as F‐ILs@HSNRs/PEO‐LiTFSI, //F‐ILs@HSNRs/PEO‐LiTFSI, and ⊥F‐ILs@HSNRs/PEO‐LiTFSI, respectively. CR2025 coin cell housings, 0.5 mm thick nickel foam, and 0.2 mm thick steel sheets were used. The batteries were pressurized and sealed, and all assembly processes were performed in a high‐purity argon glove box to prevent contamination from impurities and oxygen.

### 2.3 Characterization and Testing

X‐ray diffraction (XRD, Empyrean, Panalytical Instruments, Netherlands) was employed for phase identification, using a copper target at 40 kV, scanning speed of 5° min^−1^, and 2*θ* range of 5–90°. A Tensor 2 Fourier transform infrared (FT‐IR) spectrometer was used to qualitatively analyze chemical bonds or functional group vibrations in F‐SNRs and F‐ILs@SNRs, with a wavenumber range of 400–4000 cm^−1^ at room temperature. Scanning electron microscopy (SEM, S‐3400N, Hitachi, Japan), JSM‐IT800 field emission scanning electron microscopy, and JEM‐F200 transmission electron microscopy (TEM, Tecnai G2 20, FEI, USA) were used to observe the microstructure of HSNRs, F‐HSNRs, and F‐ILs@HSNRs. The elemental composition was detected using an energy spectrometer (Mapping). Focused ion beam‐scanning electron microscopy (FIB‐SEM) was used to observe the CSPE, with sample processing to analyze the composite solid electrolyte. The specific surface area of F‐HSNRs, before and after IL filling, was characterized using BET analysis after degassing the sample at 150 °C for 24 h. X‐ray photoelectron spectroscopy (XPS, PHI5000VersaProbe, ULVAC‐PHI, Japan) was performed using a VG ESCA‐LAB MK‐II electron spectrometer from V.G. Scientific Ltd.

The lithium‐ion conductivity of CSPE was measured at 60 °C using an electrochemical workstation (CHI660, Shanghai Chenhua Instrument Co., Ltd.) at a perturbation voltage of 10 mV and a frequency range of 10 Hz to 1 MHz. First, CSPE and stainless‐steel (SS) electrodes were assembled into a SS|CSPE|SS battery. To ensure good contact between the SS electrodes and CSPE, the battery was held at 45 °C for 30 min before conducting the impedance tests. The bulk resistance R_b_ of CSPEs was obtained from the test results of the impedance curves. The lithium‐ion conductivity σ of CSPE was then calculated using the following equation:^[^ ^36^ ^]^

(1) $$\sigma =\frac{d}{S\times R_{b}}\;$$

where *S* is the effective contact area between CSPE and SS, and *d* and *R_b_* are the thickness and internal resistance of the CSPE, respectively.

The internal impedance of LiFePO_4_|CSPE|Li and Li|CSPE|Li batteries was also measured using the electrochemical workstation at a perturbation voltage of 10 mV and a frequency range of 10 Hz to 1 MHz. The batteries with PEO‐LiTFSI CSPE, F‐HSNRs/PEO‐LiTFSI, F‐ILs@HSNRs/PEO‐LiTFSI, ∥F‐ILs@HSNRs/PEO‐LiTFSI, and ⊥F‐ILs@HSNRs/PEO‐LiTFSI were measured using a 10 mV DC pulse at 60 °C for 10 800 s. The DC polarization curves and their AC impedance plots before and after polarization were obtained. The lithium‐ion migration number t_Li+_ of the CSPE was then calculated using the following equation:^[^ ^36^ ^]^

(2) $$t_{\mathrm{Li}+}=\frac{\left(I_{s}\;\times R_{b}^{0}\right)}{\left(I_{0}\;\times R_{b}^{s}\right)}\frac{\left(\Delta V-I_{0}R_{1}^{0}\right)}{\Delta V-I_{s}R_{1}^{s}}$$

where ∆*V* is the applied voltage; *I_0_* and *Is* are the initial current and steady‐state current obtained from the direct current polarization test, respectively; *R_b_^0^* and *R_b_^s^* are the internal resistances of the CSPE before and after direct current polarization, respectively; *R_1_^0^* and *R_1_^s^* are the interfacial resistances before and after DC polarization, respectively. The higher the t_Li+_, the less anion migration, the greater the reduction in the differential concentration polarization during the charging‐discharging process, and the faster the charging‐discharging of the battery.

The galvanostatic charge‐discharge (GCD) cycling and rate performances of Li|CSPE|Li and LiFePO_4_|CSPE|Li batteries were tested at 60 °C using a battery test system (LandCT2001A, Wuhan Landian Testing Equipment Co., Ltd.). The current density for lithium symmetric batteries was 0.1 mA cm^−2^ during GCD testing and ranged from 0.02–0.5 mA cm^−2^ during rate performance testing. The GCD test for LiFePO_4_|CSPE|Li batteries was conducted at 0.1 C, with the rate performance tested in the range of 0.1–1 C. The charge‐discharge cut‐off voltages for LiFePO_4_|CSPE|Li batteries were set at 3.75 and 2.45 V, respectively.

### 2.4 Multi‐Physics Simulation

COMSOL Multiphysics 5.6 software, running on macOS Ventura with an Apple M1 Pro 10‐core CPU and 32 GB memory, was used for the simulations. The single particle conductor interface was applied to simulate the current density distribution in the constant phase element (CPE) during the charge–discharge process of the Li|CSPE|Li battery.

The CSPE simulation structure comprised a polymer electrolyte and a 3.04 µm aligned nanowire array positioned between the top and bottom lithium metal electrodes. The F‐ILs@HSNRs nanowires had a diameter of 20 nm, length of 1 µm, and spacing of 136 nm. The metal electrodes had a conductivity of 4.56 × 10^−7^ S cm^−1^ and the conductivity of the polymer electrolyte was derived from experimental values. The bottom edge of the lower electrode was grounded, while a constant input voltage of 50 mV was applied to the top edge of the upper metal electrode. All other edges were treated as electrical insulators and the initial potential in all domains was set to 0 V.

## 3 Results and Discussion

**Figure 2:**
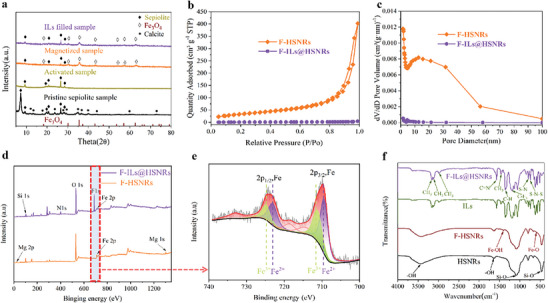
a) XRD patterns of pristine sepiolite ore, acid‐activated, magnetized, and ILs‐injected samples. b) N_2_ adsorption–desorption isotherms and c) pore size distribution of F‐HSNRs and F‐ILs@HSNRs. d) XPS survey spectra of F‐HSNRs and F‐ILs@HSNRs. e) Fe *2p* XPS fitting spectra of F‐ILs@HSNRs for the red dotted area in (d). f) FT‐IR spectra of HSNRs, F‐HSNRs, ILs, and F‐ILs@HSNRs.

The preparation process of the CSPE with aligned 1D HSNRs containing confined ILs (F‐ILs@HSNRs) is illustrated in Figure 1a. The pristine sepiolite ore sample comprised nano‐flakes and nanorods (Figure 1b). According to the XRD pattern analysis in **Figure** **2**a, the flakes were calcite impurities, while the rods were sepiolite. First, the flaky calcites in the sepiolite were removed by microwave‐assisted acid activation to obtain HSNRs. After acid activation, only nanorods remained in the sample (Figure 1c), with lengths of 300–700 nm and diameters of 25–60 nm. Subsequently, Fe_3_O_4_ magnetic particles were attached to the nanorods using a solution coating method to obtain F‐HSNRs.

Numerous nanoparticles were attached to the nanorods after magnetization (Figures 1d,e). Figure 1e shows the morphology of the magnetized nanorods, with the inset illustrating a hollow single magnetized nanorod, measuring 25∼60 nm in diameter, with black nanoparticles attached to the surface. Silicon (Si) and iron (Fe) element mapping in Figure 1e1,e2 shows that the distribution regions of Si and Fe almost coincided, confirming that the hollow nanorods were Si nanorods, with Fe_3_O_4_ magnetic particles uniformly attached, aiding the alignment of HSNRs in the PEO‐LiTFSI matrix. Subsequently, ILs were injected into the channels of F‐HSNRs using supercritical CO_2_ fluid, resulting in the confinement of ILs within HSNRs (F‐ILs@HSNRs).

Figure 1f shows the morphology after ILs injection. The inset shows a significantly darker contrast in the central channel of the F‐HSNRs, likely caused by ILs filling the nanochannels. Si and Fe element mapping in Figure 1e3,e4,f3,f4 represents the distribution of Si and Fe elements from ILs (C_8_H_11_F_6_N_3_O_4_S_2_) in the samples before and after IL injection, confirming the successful confinement of ILs within the nanochannels of F‐HSNRs. Finally, F‐ILs@HSNRs were compounded with the PEO‐LiTFSI polymer electrolyte and aligned in the CSPE matrix using a magnetic field. The CSPE membrane and electrodes were then assembled into Li|CSPE|Li and LiFePO_4_|CSPE|Li batteries.

Figure 2a presents the XRD patterns of the pristine sepiolite ore sample before and after microwave‐assisted acid activation, magnetization, and ILs injection. The sepiolite used was (Si_12_) (Mg_8_) O_30_ (OH)_4_ (OH_2_)_4_·8H_2_O, which is a hydrated Mg silicate clay mineral and a natural mineral fiber with a layered chain‐like crystal structure, consisting of two layers of Si‐O tetrahedron sandwiched by a layer of Mg‐O octahedron. The upper and lower Mg‐O octahedron layers were continuous and ran through the entire layer of chain‐like crystal. Each Si‐O tetrahedron shared two vertices and was connected with three adjacent tetrahedrons. The reactive O species in the Si‐O tetrahedron indicated periodic inversions along the b‐axis, forming an open channel of a fixed size parallel to the layered chain crystal. Therefore, the sepiolite structure contained Si‐OH, which is a hydroxyl group of crystal water and Mg‐OH.

The characteristic diffraction peaks of calcite impurities were found at 2*θ* = 11.8° (130), 13.3° (200), 17.7° (150), and 23.7° (221) for the XRD pattern of the pristine sepiolite sample in Figure 2a.^[^ ^37^ ^]^ The 2*θ* of 7.3° was attributed to the typical diffraction peak of the Mg‐O octahedron structure in the internal channels of the layered chain crystals of sepiolite. The peaks at 2*θ* = 10.7° and 26.6° (400) were ascribed to the Si‐O tetrahedron of sepiolite crystals. The peaks of calcite impurities at 2*θ* = 11.8° (130), 13.3° (200), 17.7° (150), and 23.7° (221) disappeared after microwave‐assisted acid activation, indicating that acid activation effectively removed calcite impurities in pristine sepiolite. The 2*θ* of the diffraction peak of the Mg‐O octahedron (110) at 7.3° also disappeared, indicating the removal of the Mg‐O octahedron in sepiolite. Meanwhile, the 2θ of other peaks of the Si‐O tetrahedron at 10.7° and 26.6° did not change significantly, indicating that the structure and crystallinity of the Si‐O tetrahedrons remain unchanged.^[^ ^38^ ^]^

The peaks of the Si‐O tetrahedron at 2*θ* = 26.6° were enhanced, indicating that Si‐O tetrahedrons in sepiolite crystals were purified. This was because, during the microwave‐assisted acid activation, the acid first reacted with calcite and other impurities, resulting in the disappearance of the calcite absorption peak. Subsequently, H^+^ preferentially replaced the terminal octahedral coordination Mg^2^^+^ and gradually removed the Mg^2^⁺ in the other Mg‐O octahedrons, and the Si‐O‐Mg‐O‐Si structure was transformed into two Si‐OH,^[^ ^39^ ^]^ which were carried away by the solution, thus leaving the Si‐O tetrahedron skeleton. Therefore, not only were calcite flakes removed, but pristine sepiolite was purified by microwave‐assisted acid activation treatment. Significantly, the removal of Mg^2^^+^ in the internal channels of the layered chain crystal of sepiolite, leaving a Si‐O tetrahedron skeleton, successfully prepared HSNRs. Simultaneously, the acid activation treatment resulted in the removal of Mg^2^⁺ and the formation of new pore structures,^[^ ^40^, ^41^ ^]^ which provided ample space for the injection of ILs into the internal channels of HSNRs in subsequent steps.

The main phases present in the sample after magnetization were HSNRs and Fe_3_O_4_. The XRD patterns, along with the morphological and elemental mapping analyses, confirmed the successful preparation of Fe_3_O_4_‐attached HSNRs (F‐HSNRs) using the solution method. The XRD peaks of the samples after ILs injection did not show significant changes compared to those before filling, indicating that IL injection did not alter the crystal structure of F‐HSNRs.

The N_2_ adsorption–desorption isotherms and pore size distributions of the samples before and after ILs injection are depicted in Figure 2b,c. The adsorption and desorption curves for F‐HSNRs (Figure 2b) exhibited a H_3_‐type hysteresis loop, indicative of the irregular shape of the central channel of F‐HSNRs. Figure 2c illustrates that the aperture distribution of F‐HSNRs primarily fell within the mesoporous range, with an average aperture of 17.5 nm. The specific surface area of F‐HSNRs decreased sharply from 156.98 m^2^/g before ILs injection to 2.17 m^2^/g after IL injection, while the total pore volume also decreased from 0.705875 to 0.006543 cm^3^/g, confirming that ILs successfully occupied the central channel of F‐HSNRs.

Figure 2d,e show the XPS full spectrum and Fe *2p* XPS fitting spectra of F‐HSNRs and F‐ILs@HSNRs. From Figure 2d, the primary elements in magnetized F‐HSNRs were Si and O, with trace amounts of Mg and Fe detected. After IL injection, the N 1s peak corresponding to the EMI cation of F‐ILs@HSNRs appeared at ≈399 eV. For TFSI anions, the F 1s peak was observed at ≈686 eV, further confirming that ILs were successfully injected into F‐HSNRs. Figure 2e shows the Fe *2p* XPS fitting spectra of F‐HSNRs, where the peaks at 709.51 and 722.88 eV corresponded to Fe^2+^ *2p*1/2 and *2p*3/2, respectively, while the peaks at 711.38 and 724.98 eV were attributed to Fe^3+^ *2p*1/2 and *2p*3/2. This indicates the presence of both divalent and trivalent Fe, consistent with the valence state of Fe in Fe_3_O_4_. These findings confirm that the surface of F‐HSNRs was successfully attached to Fe_3_O_4_ particles and that this coating remained unaffected after IL injection.

Figure 2f presents the FT‐IR spectra of HSNRs, F‐HSNRs, ILs, and F‐ILs@HSNRs. The FT‐IR spectrum of F‐HSNRs revealed absorption peaks at 587 and 1410 cm^−1^, attributed to the stretching vibration of Fe‐O and the hydroxyl bending (O‐H) vibration of Fe_3_O_4_, respectively.^[^ ^42^, ^43^, ^44^ ^]^ These indicate that a stable interaction was established between Fe₃O₄ nanoparticles and HSNRs via van der Waals forces. However, at the nanoscale, they agglomerated when mixed, rather than Fe₃O₄ being uniformly coated on HSNRs. In addition, Fe₃O₄ magnetic particles prepared here were mostly nanospheres and spontaneously agglomerated.

The FT‐IR spectrum of F‐ILs@HSNRs shows the characteristic vibration peaks associated with ILs. The C‐H plane bending vibration in the imidazole ring was observed at 1357 cm^−1^. The deformation vibration absorption peak of the methylene group on the imidazole ring side chain was observed at 1468 cm^−1^; the stretching vibration absorption peak of the imidazole ring skeleton was observed at 1579 cm^−1^; and the anti‐symmetric stretching vibrations of ‐CH_3_ in the imidazole cation were observed at 3169, 3126, and 2998 cm^−1^. The asymmetric in‐plane deformation of ‐CF_3_ in TFSI‐, the stretching vibration of the S‐N bond/‐CF_3_ symmetric in‐plane deformation, and the stretching vibration of the S‐N‐S bond were observed at 1191, 787, and 738 cm^−1^, respectively.^[^ ^45^, ^46^ ^]^

Notably, after IL injection, the OH vibration peaks associated with sepiolite water and coordinated water in F‐ILs@HSNRs at 1637 and 3436 cm^−1^ were significantly weakened, indicating that ILs entered the central channel of F‐HSNRs, replacing some of the sepiolite water.^[^ ^47^, ^48^ ^]^ The coordination water was removed in the process of acid activation. H^+^ preferentially replaced the terminal octahedral coordination Mg^2^^+^ and gradually removed the Mg^2^⁺ in the other Mg‐O octahedrons during microwave‐assisted acid activation. The two coordination water molecules bound to Mg^2^⁺ were carried away together with Mg^2^⁺ by solution and then removed by evaporation when dried at 105 °C.

**Figure 3:**
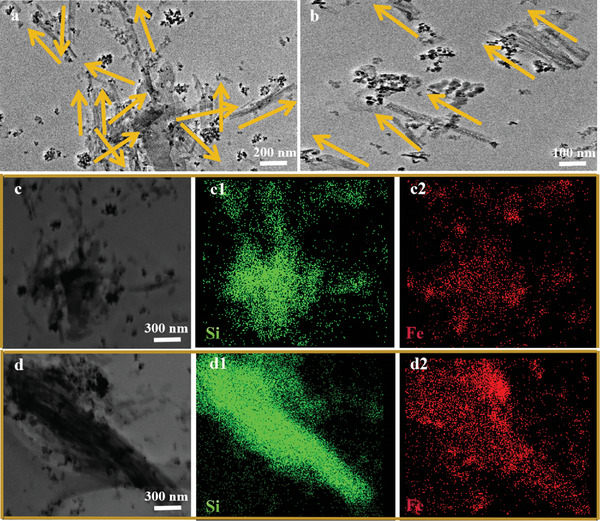
TEM images of CSPE prepared under a) unapplied and b) applied magnetic fields. TEM images and Si and Fe element mapping of CSPE prepared under c–c2) unapplied and d–d2) applied magnetic fields.

The TEM images and the distribution of Si and Fe elements in the CSPE samples prepared under unapplied and applied magnetic fields are shown in **Figure** **3**. Based on the analysis of Figure 1e,h, the nanorods in the TEM images of Figure 3 represent F‐ILs@HSNRs, while the black nanoparticles are Fe_3_O_4_. In the CSPE samples prepared without the application of a magnetic field (Figure 3a), F‐ILs@HSNRs exhibited a disordered arrangement. In contrast, in the CSPE samples obtained under the application of a magnetic field (Figure 3b), F‐ILs@HSNRs were aligned in a uniform direction. The element mappings in Figure 3c–c2,d–d2 further support this observation. On this basis, the orientation of F‐ILs@HSNRs in the CSPE was parallel or perpendicular to the electrode sheets during the assembly of solid‐state lithium batteries.

**Figure 4:**
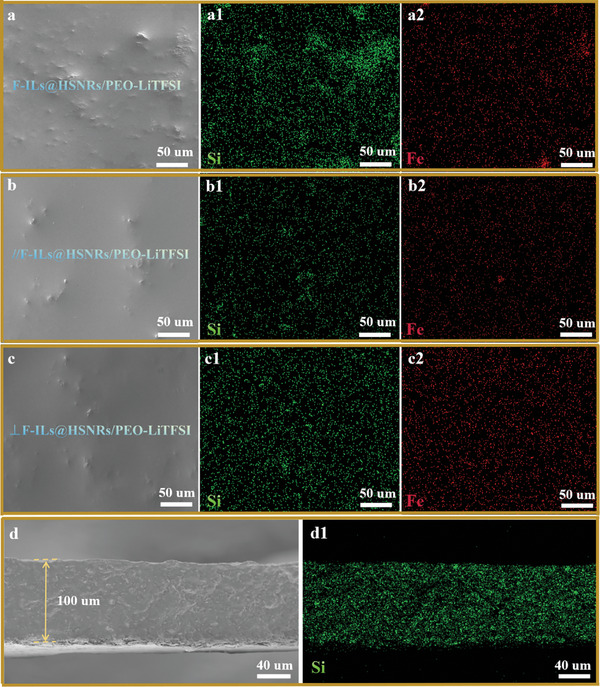
SEM images and Si and Fe element mapping of the circular upper surfaces on cylindrical samples of a–a2) F‐ILs@HSNRs/PEO‐LiTFSI, b–b2) //F‐ILs@HSNRs/PEO‐LiTFSI, and c–c2) ⊥F‐ILs@HSNRs/PEO‐LiTFSI. SEM images and Si element mapping of the cross‐section of d,d1) ⊥F‐ILs@HSNRs/PEO‐LiTFSI.

**Figure** **4** presents the SEM images and distribution of Si and Fe elements in CSPE samples obtained under both unapplied and applied magnetic fields. Compared to the distribution of Si elements in the CSPE samples prepared without a magnetic field (Figure 4a–a2), the Si element distribution in samples prepared under a magnetic field (Figure 4b–b2,c–c2) was more uniform, regardless of whether they were //F‐ILs@HSNRs/PEO‐LiTFSI or ⊥F‐ILs@HSNRs/PEO‐LiTFSI. This indicates that the application of a magnetic field promoted the uniform dispersion of magnetic F‐ILs@HSNRs within the PEO‐LiTFSI polymer electrolyte matrix.

From the SEM image and Si mapping of the cross‐section of ⊥F‐ILs@HSNRs/PEO‐LiTFSI in Figure 4d,d1, the thicknesses of the CSPE films of ⊥F‐ILs@HSNRs/PEO‐LiTFSI were consistent, at ≈100 µm, and the Si elements were evenly distributed in the thickness direction. This indicates that F‐ILs@HSNRs were evenly distributed in the thickness direction of the CSPE films of ⊥F‐ILs@HSNRs/PEO‐LiTFSI. This improves the overall lithium‐ion conduction capacity of CSPEs and the overall energy density of batteries.

**Figure 5:**
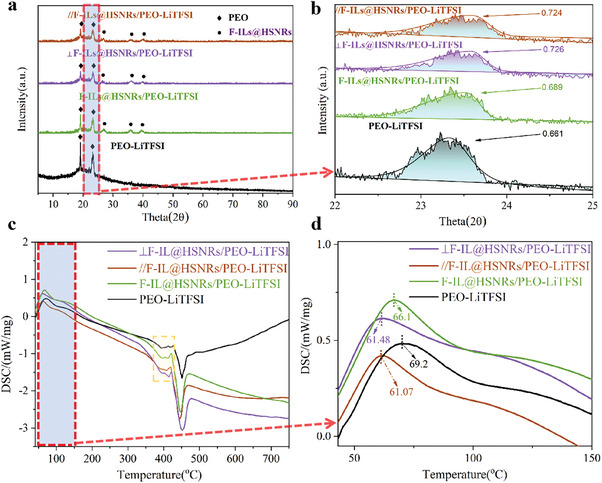
a) XRD patterns and b) full width at half maximum (FWHM) of the diffraction peaks in the range 22.5‐24.3°. DSC curves for PEO‐LiTFSI CSPE, F‐ILs@HSNRs/PEO‐LiTFSI, ∥F‐ILs@HSNRs/PEO‐LiTFSI, and ⊥F‐ILs@HSNRs/PEO‐LiTFSI in the temperature ranges c) 40–750 °C and d) 40–150 °C.

As shown in the XRD pattern of the CSPE in **Figure** **5**a, two prominent characteristic peaks can be observed at 19.5 and 23.5°, corresponding to the crystallization region of PEO. The diffraction peaks for the three CSPE samples containing 15% F‐ILs@HSNRs were primarily attributed to F‐ILs@HSNRs and PEO. Furthermore, as indicated in Figure 5b, the full width at half maximum (FWHM) of the diffraction peak in the range 22.5–24.3° increased from 0.661–0.689, suggesting that the inclusion of F‐ILs@HSNRs reduced the crystallinity of PEO, which facilitated the movement of PEO chain segments and enhanced lithium‐ion transport. Additionally, the FWHM values for the diffraction peaks of ∥F‐ILs@HSNRs/PEO‐LiTFSI and ⊥F‐ILs@HSNRs/PEO‐LiTFSI were 0.724 and 0.726, respectively, compared to 0.689 for the randomly arranged F‐ILs@HSNRs/PEO‐LiTFSI, indicating that the orientation of F‐ILs@HSNRs in the CSPE affected PEO crystallinity.

The DSC curves of the CSPE are presented in Figure 5c,d. The exothermic peaks of the CSPE with F‐ILs@HSNRs shifted to lower temperatures. The melting points of F‐ILs@HSNRs/PEO‐LiTFSI, ∥F‐ILs@HSNRs/PEO‐LiTFSI, and ⊥F‐ILs@HSNRs/PEO‐LiTFSI decreased from 69.2 °C for PEO‐LiTFSI to 66.1, 61.481, and 61.07 °C, respectively, due to the reduced crystallinity of PEO upon the incorporation of F‐HSNRs. Moreover, the exothermic peak area of the CSPE with F‐ILs@HSNRs was significantly larger than that of PEO‐LiTFSI in the temperature range 350–430 °C, primarily due to the volatilization of ILs. Additionally, the exothermic peak areas of ∥F‐ILs@HSNRs/PEO‐LiTFSI and ⊥F‐ILs@HSNRs/PEO‐LiTFSI were greater than that of the randomly arranged F‐ILs@HSNRs/PEO‐LiTFSI, which was attributed to the more uniform dispersion of F‐ILs@HSNRs resulting from the application of a magnetic field (detailed explanation in Section S2, Supporting Information), thereby enhancing the IL volatilization.

The thermal decomposition of LiTFSI and PEO occurred within the same temperature range, with both starting to decompose at ≈400 °C. The exothermic peak observed at 440 °C was primarily related to thermal decomposition.

**Figure 6:**
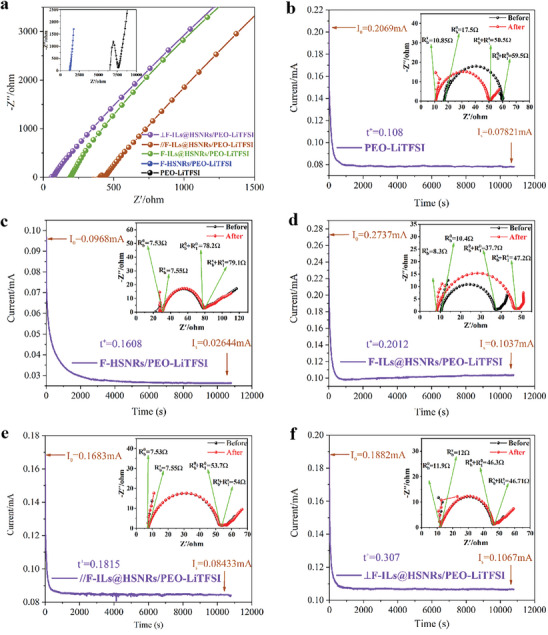
a) Room‐temperature impedance plots of PEO‐LiTFSI CSPE (inset), F‐HSNRs/PEO‐LiTFSI (inset), F‐ILs@HSNRs/PEO‐LiTFSI, ∥F‐ILs@HSNRs/PEO‐LiTFSI, and ⊥F‐ILs@HSNRs/PEO‐LiTFSI. b–f) DC polarization curves of Li|CSPE|Li batteries using PEO‐LiTFSI CSPE, F‐HSNRs/PEO‐LiTFSI, F‐ILs@HSNRs/PEO‐LiTFSI, ∥F‐ILs@HSNRs/PEO‐LiTFSI, and ⊥F‐ILs@HSNRs/PEO‐LiTFSI, along with their corresponding AC impedance plots before and after polarization (insets).

The room‐temperature impedance of the CSPE is shown in **Figure** **6**a. From high to low, the order was: PEO‐LiTFSI < F‐HSNRs/PEO‐LiTFSI < ∥F‐ILs@HSNRs/PEO‐LiTFSI < F‐ILs@HSNRs/PEO‐LiTFSI < ⊥F‐ILs@HSNRs/PEO‐LiTFSI. To evaluate the lithium‐ion migration number t_Li+_ of the CSPE, the DC polarization curves and their corresponding AC impedance plots before and after polarization for Li|CSPE|Li batteries using PEO‐LiTFSI CSPE, F‐HSNRs/PEO‐LiTFSI, F‐ILs@HSNRs/PEO‐LiTFSI, ∥F‐ILs@HSNRs/PEO‐LiTFSI, and ⊥F‐ILs@HSNRs/PEO‐LiTFSI were recorded using a 10 mV DC pulse at 60 °C for 10800 s. The DC polarization curves and their AC impedance plots before and after polarization were also obtained. The lithium‐ion migration number t_Li+_ of the CSPE calculated according to Equation (2) is shown in Figure 6b–f. A higher t_Li+_ indicates a lower number of anion migrations, which is advantageous for reducing differential concentration polarization during the charging and discharging processes, thereby enhancing the charging and discharging rates of the battery.

The calculated t_Li+_ values followed the same order as the room‐temperature impedance. The conductivity and t_Li+_ of the PEO‐LiTFSI CSPE were the lowest, measured at 1.75 × 10^−6^ S cm^−1^ and 0.108, respectively. With the addition of F‐HSNRs, the conductivity of F‐HSNRs/PEO‐LiTFSI increased to 1.23 × 10^−5^ S cm^−1^, ≈7.1 times higher, and t_Li+_ increased to 0.168. As F‐HSNRs did not conduct Li^+^, the enhancement was attributed to two factors: first, the incorporation of F‐HSNRs decreased the crystallinity of PEO; second, Li^+^ transport pathways were formed at the interface between F‐HSNRs and the PEO‐LiTFSI matrix, improving lithium‐ion transmission efficiency.

With the addition of F‐ILs@HSNRs, the conductivity (6.52 × 10^−5^ S cm^−1^) and t_Li+_ (0.2012) of F‐ILs@HSNRs/PEO‐LiTFSI were 37 times and 86% higher than those of PEO‐LiTFSI, respectively. This significant increase was primarily due to the ILs being confined to the central channels of F‐HSNRs, providing 1D fast pathways for Li^+^ transport and significantly enhancing the conductivity of the CSPE. Additionally, the Si‐O bonds of F‐HSNRs at the interface between F‐HSNRs and the PEO‐LiTFSI matrix contributed to the dissociation of Li⁺TFSI⁻ ion pairs through several mechanisms involving coordination actions, electric field depletion, and competitive adsorption, thereby increasing the concentration of free Li⁺, forming the Li⁺ transport pathways^[^ ^49^ ^]^ (detailed explanation in Section S3, Supporting Information).

The orientation of F‐ILs@HSNRs significantly affected the room‐temperature impedance and t_Li+_ of the CSPE. The conductivity (2.14 × 10^−4^ S·cm^−1^) and t_Li+_ (0.307) of ⊥F‐ILs@HSNRs/PEO‐LiTFSI were the highest, representing 125 times and 184% increases compared to PEO‐LiTFSI, respectively. In contrast, the conductivity and t_Li+_ of ∥F‐ILs@HSNRs/PEO‐LiTFSI were only 3.37 × 10^−5^ S cm^−1^ and 0.182, respectively, which were even lower than those of F‐ILs@HSNRs/PEO‐LiTFSI.

**Figure 7:**
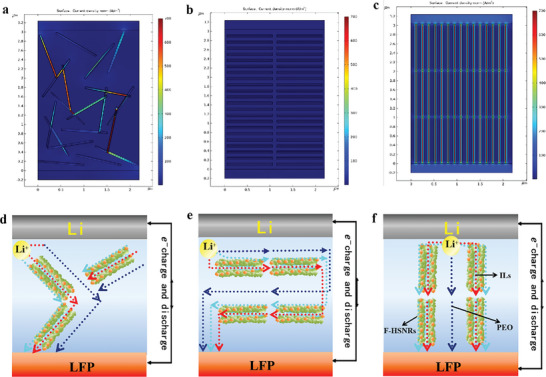
Schematic illustrations of lithium‐ion transport in a) F‐ILs@HSNRs/PEO‐LiTFSI, b) ∥F‐ILs@HSNRs/PEO‐LiTFSI, and c) ⊥F‐ILs@HSNRs/PEO‐LiTFSI. Simulated current density distributions are shown in d) F‐ILs@HSNRs/PEO‐LiTFSI, e) ∥F‐ILs@HSNRs/PEO‐LiTFSI, and f) ⊥F‐ILs@HSNRs/PEO‐LiTFSI.

To ascertain the mechanism of the observed phenomenon, Comsol Multiphysics software was employed to simulate the charging‐discharging process of Li|CSPE|Li batteries, resulting in the current density distribution within the CSPE, as illustrated in **Figure** **7**a–c. The calculated electrical conductivity of the ILs in F‐ILs@HSNRs and ILs@HSNRs/PEO interfaces was 5.4 × 10^−3^ S cm^−1^ and 9.6 × 10^−2^ S cm^−1^, respectively, which is generally consistent with the lithium‐ion conductivity reported in the literature.^[^ ^50^, ^51^ ^]^ The conductivity of ILs in F‐ILs@HSNRs was an order of magnitude higher than that at the ILs@HSNRs/PEO interfaces and two orders of magnitude higher than the total conductivity, indicating that the fast pathways created by ILs in F‐ILs@HSNRs significantly enhanced the transmission efficiency of Li^+^.

As shown in Figure 7c, the total conductivity of ⊥F‐ILs@HSNRs/PEO‐LiTFSI was 2.14 × 10^−4^ S cm^−1^ (at 30 °C), closely matching the experimental data of 2.03 × 10^−4^ S cm^−1^. The highest current density was observed in the ILs of F‐ILs@HSNRs positioned perpendicular to the electrodes, while the second highest current density was observed at the ILs@HSNRs/PEO‐LiTFSI interface, and the lowest current density was within the PEO‐LiTFSI matrix. Figure 7a demonstrates that the total conductivity of F‐ILs@HSNRs/PEO‐LiTFSI decreased to 7.52×10^−5^ S cm^−1^ with its current density distribution significantly lower than that of ⊥F‐ILs@HSNRs/PEO‐LiTFSI.

Within the disordered F‐ILs@HSNRs, the ILs in the central channels exhibited the highest current density, while the lowest current density remained in the PEO‐LiTFSI matrix. The Li^+^ migration pathways formed by ILs in F‐ILs@HSNRs with high current density were tortuous and considerably longer than those in ⊥F‐ILs@HSNRs/PEO‐LiTFSI. In Figure 7b, the total conductivity of ∥F‐ILs@HSNRs/PEO‐LiTFSI further declined to 3.37 × 10^−5^ S cm^−1^, and the overall current density was low. The current density at the ILs@HSNRs/PEO‐LiTFSI interfaces and the ILs in F‐ILs@HSNRs parallel to the electrodes was only slightly higher than that of the PEO‐LiTFSI matrix.

Based on the simulation results, schematics of the Li^+^ transport paths in F‐ILs@HSNRs/PEO‐LiTFSI, ∥F‐ILs@HSNRs/PEO‐LiTFSI, and ⊥F‐ILs@HSNRs/PEO‐LiTFSI were constructed, as shown in Figure 7d–f. The significant influence of the arrangement direction of F‐ILs@HSNRs on the room‐temperature impedance and t_Li+_ of CSPE was attributed to the ILs confined in F‐HSNRs, which provided 1D fast pathways for Li⁺ transmission, thereby significantly enhancing the Li^+^ conductivity of the CSPE. The migration paths of Li^+^ in CSPE, ranked from shortest to longest, were as follows: ⊥F‐ILs@HSNRs/PEO‐LiTFSI < F‐ILs@HSNRs/PEO‐LiTFSI < ∥F‐ILs@HSNRs/PEO‐LiTFSI.

As shown in Figure 7f, the F‐ILs@HSNRs in ⊥F‐ILs@HSNRs/PEO‐LiTFSI were uniformly dispersed and arranged perpendicular to the electrodes, providing the shortest migration paths for Li^+^ and minimizing the potential barrier to Li^+^ migration. These factors contributed to the highest Li^+^ transmission efficiency, resulting in excellent Li^+^ conductivity. Although F‐ILs@HSNRs were evenly dispersed in ∥F‐ILs@HSNRs/PEO‐LiTFSI, as illustrated in Figure 7e, their parallel arrangement to the electrodes led to the longest migration paths for Li^+^. Consequently, the Li^+^ conductivity of ∥F‐ILs@HSNRs/PEO‐LiTFSI was lower than that of F‐ILs@HSNRs/PEO‐LiTFSI (Figure 7d).

**Figure 8:**
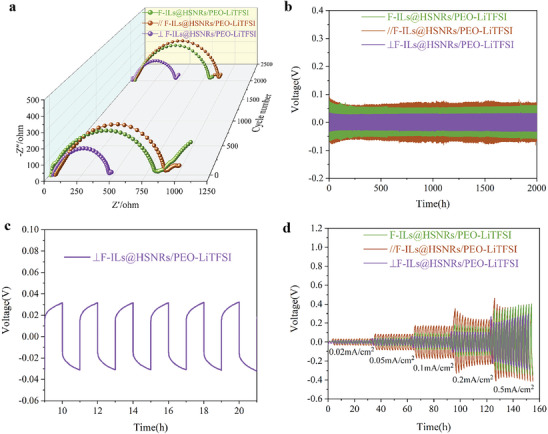
a) Impedance before and after GCD cycling at 0.1 mA cm^−2^ at 60 °C, b,c) polarization voltage during GCD cycling at 0.1 mA cm^−2^, and d) rate performance of GCD cycling at 0.02 to 0.5 mA cm^−2^ for Li|CSPE|Li batteries with F‐ILs@HSNRs/PEO‐LiTFSI,∥F‐ILs@HSNRs/PEO‐LiTFSI, and ⊥F‐ILs@HSNRs/PEO‐LiTFSI.

**Figure** **8**a illustrates the internal impedance characteristics before and after GCD cycling at 0.1 mA cm^−2^ at 60 °C for Li|CSPE|Li batteries utilizing F‐ILs@HSNRs/PEO‐LiTFSI, and ⊥F‐ILs@HSNRs/PEO‐LiTFSI. The observed incomplete semicircle in the high‐frequency region signified the impedance associated with CSPEs, while the intersection of the semicircle with the real axis in the low‐frequency region corresponded to the internal resistance (R_int_) of the lithium‐ion battery. Notably, the Li|CSPE|Li battery featuring ∥F‐ILs@HSNRs/PEO‐LiTFSI before and after GCD cycling exhibited the highest R_int_ of 916 and 921 Ω respectively, suggesting that the parallel orientation of F‐ILs@HSNRs was detrimental to the conductivity of the CSPE.

In contrast, the R_int_ before and after GCD cycling of the Li|CSPE|Li battery with ⊥F‐ILs@HSNRs/PEO‐LiTFSI was minimized at 466 and 471 Ω respectively, significantly lower than that of the batteries with F‐ILs@HSNRs/PEO‐LiTFSI (816 Ω, 819 Ω) and ∥F‐ILs@HSNRs/PEO‐LiTFSI configurations. The R_int_ of Li‐ion batteries was predominantly influenced by the bulk resistance of the CSPEs and the interfacial resistance between the CSPEs and lithium electrodes. The minimized R_int_ observed in the ⊥F‐ILs@HSNRs/PEO‐LiTFSI configuration was attributable to its reduced interfacial resistance. This was corroborated by the data presented in Figure 7d–f, which indicated that the current density at the interface between ⊥F‐ILs@HSNRs/PEO‐LiTFSI and the lithium electrodes, as depicted in Figure 7f, was the highest, thereby suggesting an enlarged effective contact area that mitigated interfacial resistance.

Figure 8b depicts the GCD cycling performance of the Li|CSPE|Li batteries. Under GCD cycling at a current density of 0.1 mA cm^−2^, the initial polarization voltages for the F‐ILs@HSNRs/PEO‐LiTFSI and ∥F‐ILs@HSNRs/PEO‐LiTFSI batteries were recorded at 81.6 and 91.6 mV, respectively. After 2000 cycles, the polarization voltages stabilized at ≈62.1 and 71.8 mV, respectively. Conversely, the ⊥F‐ILs@HSNRs/PEO LiTFSI battery maintained a consistent polarization voltage of ≈31.6 mV throughout the GCD cycling, indicating that the perpendicular arrangement of F‐ILs@HSNRs relative to the electrodes enhanced the Li^+^ migration rate within the electrolyte membrane. As demonstrated in Figure 8c, the stable polarization voltage noted during the initial cycles further suggested improved compatibility between ⊥F‐ILs@HSNRs/PEO‐LiTFSI and the lithium electrodes, contributing to a more stable spatial electric field.

Figure 8d presents the rate performance of Li|CSPE|Li batteries with varying configurations, including F‐ILs@HSNRs/PEO‐LiTFSI, ∥F‐ILs@HSNRs/PEO‐LiTFSI, and ⊥F‐ILs@HSNRs/PEO‐LiTFSI. The batteries demonstrated robust cycling stability during GCD cycling across current densities of 0.02, 0.05, 0.1, 0.2, and 0.5 mA cm^−^^2^. The⊥F‐ILs@HSNRs/PEO‐LiTFSI battery exhibited the lowest polarization voltage, attributed to the uniform dispersion of F‐ILs@HSNRs within the CSPE and their perpendicular alignment with the electrodes, which provided Li^+^ with the shortest and most efficient migration pathways. This configuration effectively reduced the intrinsic impedance and polarization voltage of the CSPE, thereby enhancing the cycling stability.

**Figure 9:**
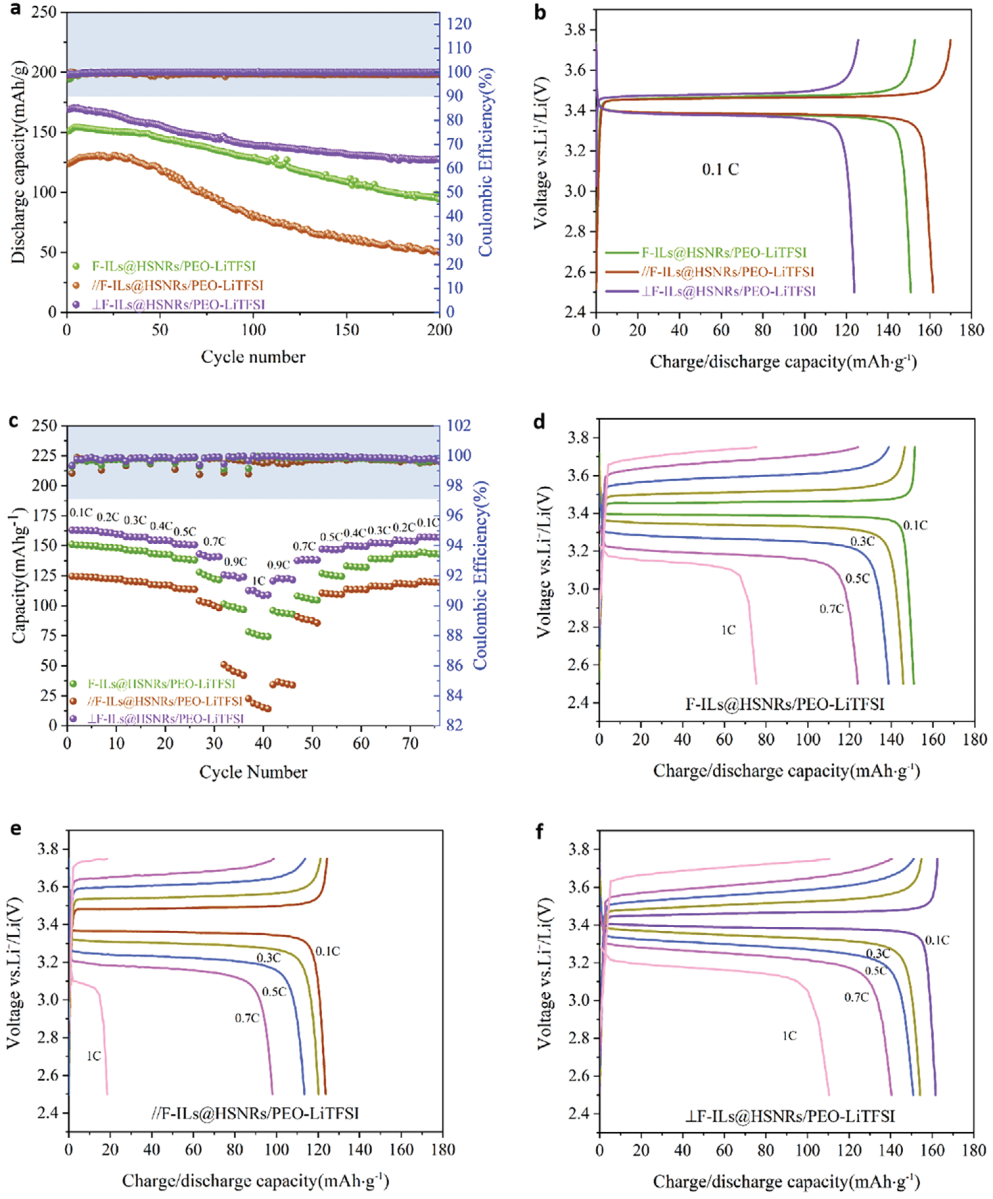
a) GCD cycling performance, b) first cycle discharge specific capacity and coulomb efficiency (light blue background), c) rate performance and coulomb efficiency (light blue background), and d–f) discharge specific capacity at different rates of LiFePO_4_|CSPE|Li batteries with F‐ILs@HSNRs/PEO‐LiTFSI,∥F‐ILs@HSNRs/PEO‐LiTFSI, and ⊥F‐ILs@HSNRs/PEO‐LiTFSI.

The GCD cycling performance and coulombic efficiency of LiFePO4|CSPE|Li batteries tested at 0.1 C and 60 °C are shown in **Figure** **9**a,b and **Table** **1**. The initial discharge specific capacities of the batteries from high to low were: ⊥F‐ILs@HSNRs/PEO‐LiTFSI > F‐ILs@HSNRs/PEO‐LiTFSI > ∥F‐ILs@HSNRs/PEO‐LiTFSI. After 200 cycles, the discharge specific capacity of the batteries decreased significantly; however, the capacity retention rate of ⊥F‐ILs@HSNRs/PEO‐LiTFS remained considerably higher than the other two, reaching ≈80%, with a Coulombic efficiency of almost 100%. Over multiple cycles, the discharge specific capacity and Coulombic efficiency of LiFePO_4_|CSPE|Li batteries were significantly reduced due to the formation and growth of lithium dendrites in the CSPE membrane during repeated charge–discharge cycles. This growth reduced the availability of free Li^+^, leading to a decreased discharge specific capacity. The ∥F‐ILs@HSNRs/PEO‐LiTFSI battery exhibited the lowest capacity retention and Coulombic efficiency because the F‐ILs@HSNRs in the CSPE membrane were arranged parallel to the electrodes, resulting in the longest migration pathways for Li^+^.

**Table 1:** GCD cycling performance and discharge specific capacity of LiFePO_4_ batteries.

		F‐ILs@HSNRs/PEO‐LiTFSI	//F‐ILs@HSNRs/PEO‐LiTFSI	⊥F‐ILs@HSNRs/PEO‐LiTFSI
Initial discharge specific capacity (mAh g^−1^)		150.8	123.81	161.65
Discharge specific capacity after 200 cycles (mAh g^−1^)		94.16	49.71	128.5
Capacity retention (%)		62.4	40.1	80
Discharge specific capacity (mAh g^−1^)	0.1 C	150.8	123.81	161.65
	0.2 C	148.3	122.3	159.91
	0.3 C	145.39	120.23	154.13
	0.4 C	142.97	116.67	153.18
	0.5 C	138.57	113.67	150.92
	0.7 C	123.29	101.13	140.39
	0.9 C	98.64	47.08	126.29
	1 C	75.14	22.09	110.43

This configuration increased the intrinsic impedance of the CSPE, rendering the membrane more susceptible to lithium dendrite formation during charge–discharge cycles. Consequently, lithium dendrites grew faster and pierced the CSPE membrane, leading to short circuits and rapid decreases in specific discharge capacity. In contrast, the ⊥F‐ILs@HSNRs/PEO‐LiTFSI battery demonstrated the highest capacity retention rate and most stable GCD cycle performance. This stability was attributed to the uniform dispersion of F‐ILs@HSNRs in the CSPE and their arrangement perpendicular to the electrodes, which provided Li^+^ with the shortest migration pathways and highest transmission efficiency. This configuration facilitated Li^+^ transmission, reduced the intrinsic impedance of the CSPE, and slowed the generation of lithium dendrites, thereby stabilizing cycle performance and extending battery life.

Figure 9c shows the rate performance of LiFePO_4_|CSPE|Li batteries at 60 °C. The charge–discharge rate increased from 0.1 to 1 C every five cycles and then returned to 0.1 C. The charge–discharge cut‐off voltages were 3.75 and 2.45 V. The discharge specific capacities at different rates are shown in Figure 9d–f and Table 1. The results indicate that LiFePO_4_|CSPE|Li batteries can be charged and discharged at the corresponding rates. Notably, the ⊥F‐ILs@HSNRs/PEO‐LiTFSI battery showed that the discharge specific capacity could be almost completely recovered when the charge–discharge rate was restored to 0.1 C, with a Coulombic efficiency of almost 100%. Moreover, its specific discharge capacity at 1 C reached 110.43 mAh g^−1^, indicating that ⊥F‐ILs@HSNRs/PEO‐LiTFSI has a good electrochemical reversibility and high specific capacity.

## 4 Conclusion

In this study, two types of solid‐state lithium batteries based on a CSPE with aligned HSNRs containing ILs were successfully developed using cost‐effective, 1D hollow natural sepiolite and ILs as raw materials, resulting in improved electrochemical performance at a low cost. The ILs were confined within the hollow channels of HSNRs and arranged perpendicular to the electrodes without leakage, enabling utilization of their excellent conductivity. This reduced the amount of ILs required and the overall cost of the battery. The application of a magnetic field enabled uniform dispersion and orientation of F‐ILs@HSNRs in CSPE. The addition of F‐ILs@HSNRs reduced the crystallinity of PEO and created Li^+^ transport pathways at the F‐ILs@HSNRs/PEO interface.

Calculated results and multi‐physics simulation analysis indicated that the ILs filled in F‐ILs@HSNRs contributed the most to lithium‐ion conduction, followed by the F‐ILs@HSNRs/PEO interface. The arrangement direction of F‐ILs@HSNRs significantly influenced the room temperature impedance and lithium‐ion conductivity of CSPE. In ⊥F‐ILs@HSNRs/PEO‐LiTFSI, the F‐ILs@HSNRs were uniformly dispersed and oriented perpendicular to the electrodes, providing the shortest and fastest migration pathways for Li^+^, thereby achieving stable and efficient lithium‐ion conduction. Compared to F‐ILs@HSNRs/PEO‐LiTFSI and ∥F‐ILs@HSNRs/PEO‐LiTFSI, the conductivity (2.14 × 10^−4^ S cm^−1^) and t_Li+_ (0.307) of ⊥F‐ILs@HSNRs/PEO‐LiTFSI were the highest, being 125 times and 184% greater than that of PEO‐LiTFSI, respectively. Moreover, its lithium‐symmetric and LiFePO_4_ batteries demonstrated stable cycling performance over 2000 h and exhibited excellent electrochemical reversibility in rate performance tests. It also achieved the smallest R_int_ (466 Ω), the lowest polarization voltage, the highest capacity retention rate, and the most stable GCD cycling performance, with a Coulombic efficiency approaching 100%.

The findings of this study broaden the material framework for solid‐state lithium batteries and provide new possibilities and solutions for the commercial application and large‐scale industrial preparation of organic/inorganic CSPE. Future studies are required to evaluate the interfacial compatibility and lithium dendrite inhibition ability of the CSPE, as well as the performances of batteries under higher currents, to further explore the relevant mechanisms.

## Conflict of Interest

The authors declare no conflict of interest.

## Supporting information



Supporting Information

## Data Availability

The data that support the findings of this study are available from the corresponding author upon reasonable request.
