# Supplementary material for: Aligned Hollow Silicon Nanorods Containing Ionic Liquid Enhanced Solid Polymer Electrolytes with Superior Cycling and Rate Performance
Source: Adv Sci (Weinh). 2024 Nov 21;12(2):2411437. doi: 10.1002/advs.202411437 (PMC11727116; doi:10.1002/advs.202411437)
Supplement: Supplementary file 1 — Supporting Information [file ADVS-12-2411437-s001.docx]

**Supporting Information**

**ALIGNED HOLLOW SILICON NANORODS CONTAINING IONIC LIQUID ENHANCED SOLID POLYMER ELECTROLYTES WITH SUPERIOR CYCLING AND RATE PERFORMANCE**

Xinglong Gao^1,2^, Zhong Zheng^1^[[1]](#footnote-1)^*^, Yifan Pan^1^, Shuyi Song^1^, Zhen Xu^2,3^

^1^ Hubei Key Laboratory of Modern Manufacturing Quantity Engineering, School of Mechanical Engineering, Hubei University of Technology, Wuhan, Hubei 430068, China

^2^ Xinjiang Key Laboratory of High Value Green Utilization of Low-Rank Coal, School of Physics and Materials Science, Changji University, Changji, Xinjiang 831100, China

^3^ School of Energy and Power Engineering, Shandong University, Qingdao, Shandong 266100, China

1.


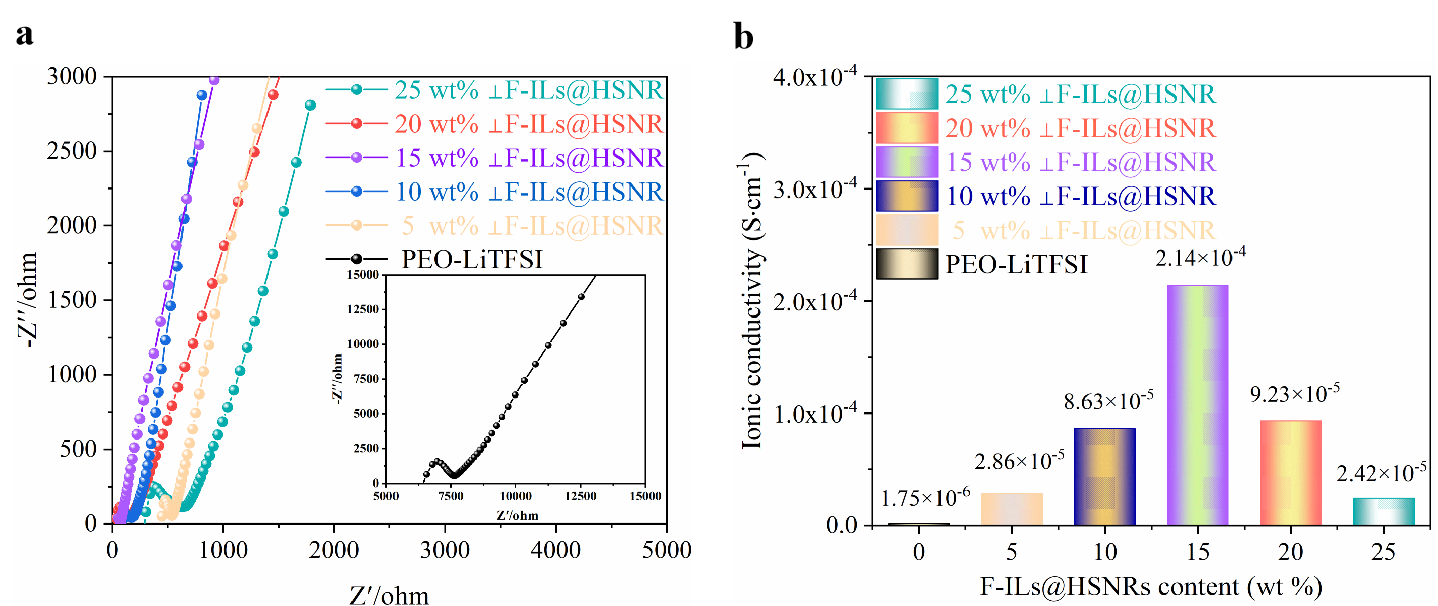


**Figure S1.** (a) Impedance plots and (b) ionic conductivity of ⊥F-ILs@HSNRs/PEO-LiTFSI with various content of F-ILs@HSNRs.

Figures S1 (a) and (b) show the effect of the weight percentage of F-ILs@HSNRs in ⊥F-ILs@HSNRs/PEO-LiTFSI on the room temperature impedance and the ionic conductivity of CSPE, respectively. With the increase of F-ILs@HSNRs, the room temperature impedance of CSPE showed a trend of decreasing first, then increasing and then decreasing, while the ionic conductivity showed an opposite trend. Among them, CSPE with 15wt % F-ILs@HSNRs had the lowest room temperature impedance and the highest ionic conductivity. The main reason why the conductivity increased with the increase of F-ILs@HSNRs before F-ILs@HSNRs was less than 15 wt% is that more ILs in F-HSNRs provided more fast channels for Li^+^ transport. However, when F-ILs@HSNRs was more than 15 wt%, the conductivity decreased with the increase of F-ILs@HSNRs, which may be due to the serious agglomeration of too many F-ILs@HSNRs in CSPE. In fact, when preparing CSPE by stirring, we also observed worse agglomeration in samples with F-ILs@HSNRs more than 15 wt%. Agglomeration may cause uneven distribution of more in the matrix, weaken the effect of magnetic field on it, and reduce the degree of directional arrangement.

2. DSC curves of CSPE is shown in **Figure 5.** (c) and (d). It can be seen that the exothermic peak area of ∥F-ILs@HSNRs/PEO-LiTFSI, and ⊥F-ILs@HSNRs/PEO-LiTFSI is larger than that of the randomly arranged F-ILs@HSNRs/PEO-LiTFSI. This is because the application of magnetic field made the dispersion of F-ILs@HSNRs more uniform.

It is considered that the dispersion of F-ILs@HSNRs had an effect on the thermal properties of composite solid polymer electrolyte (CSPE). When F-ILs@HSNRs were uniformly dispersed, CSPE tends to exhibit more consistent thermal behavior. Uniform dispersion resulted in sharper and more distinct exothermic peaks, characterized by larger and more pronounced peak areas ^[36, 51]^. This is because all F-ILs@HSNRs could fully participate in the thermal event, the heat release was more concentrated. Conversely, uneven distribution of F-ILs@HSNRs resulted in localized variations in thermal performance. This non-uniformity may lead to broadening of exothermic peaks or multiple exothermic peaks, and the peak area may become smaller or irregular. This is because F-ILs@HSNRs in some regions did not participate fully in the thermal events, or participated in an inconsistent way, resulting in a less concentrated heat release.

3. The Si-O bonds of F-HSNRs at the interface between F-HSNRs and the PEO-LiTFSI matrix contributed to the dissociation of Li⁺TFSI⁻ ion pairs through several mechanisms involving coordination actions, electric field depletion, and competitive adsorption, thereby increasing the concentration of free Li⁺, forming the Li⁺ transport pathways ^[51,53]^.

1. Coordination action: Oxygen atoms of the Si-O bonds can act as Lewis bases by forming temporary coordination bonds with Li⁺ through weak coordination actions, thereby "extracting" Li⁺ from TFSI⁻ and increasing the proportion of free Li⁺.
2. Electric field depletion: Si-O bonds exhibited strong polarity owing to asymmetric charge distribution between atoms. Oxygen atoms had a partial negative charge, and silicon atoms had a partial positive charge. This allowed the Si-O bonds to interact with TFSI⁻, diminishing the electric field of TFSI⁻, thereby weakening the electrostatic attraction between Li⁺ and TFSI⁻ and reducing the energy required for the dissociation of Li⁺TFSI⁻ ion pairs.
3. Competitive adsorption: Si-O bonds had a higher affinity for Li⁺ than for TFSI⁻, so they tended to adsorb Li⁺, further enhancing the dissociation of Li⁺ and TFSI⁻.

In general, through these mechanisms, Si-O bonds at the interface between F-HSNRs and the PEO-LiTFSI matrix effectively captured and adsorbed Li⁺, thereby contributing to the migration of Li⁺, forming the Li⁺ transmission pathways. Furthermore, the results of calculation and multi-physics simulation by COMSOL software also verified that.

1. *Corresponding author: Zhong Zheng, Tel: +86 027 59750012, Fax: +86 027 59750012, E-Mail address: zhengzh@hbut.edu.cn [↑](#footnote-ref-1)
